# Glucose and glutamine availability regulate HepG2 transcriptional responses to low oxygen

**DOI:** 10.12688/wellcomeopenres.14839.1

**Published:** 2018-09-26

**Authors:** Alvina G. Lai, Donall Forde, Wai Hoong Chang, Fang Yuan, Xiaodong Zhuang, Claudia Orbegozo Rubio, Chun-Xiao Song, Jane A. McKeating

**Affiliations:** 1 Nuffield Department of Medicine, Target Discovery Institute, University of Oxford, Oxford, OX3 7FZ, UK; 2 Ludwig Institute for Cancer Research, University of Oxford, Oxford, OX3 7DQ, UK; 3 Key Laboratory of Bioorganic Chemistry and Molecular Engineering, College of Chemistry, Peking University, Beijing, 100871, China

**Keywords:** Glucose, glutamine, HepG2, hypoxia, HIF, dioxygenases, TET, methylation, hepatocellular carcinoma

## Abstract

**Background:** Little is known about the impact of nutrients on cellular transcriptional responses, especially in face of environmental stressors such as oxygen deprivation. Hypoxia-inducible factors (HIF) coordinate the expression of genes essential for adaptation to oxygen-deprived environments. A second family of oxygen-sensing genes known as the alpha-ketoglutarate-dependent dioxygenases are also implicated in oxygen homeostasis and epigenetic regulation. The relationship between nutritional status and cellular response to hypoxia is understudied. *In vitro* cell culture systems frequently propagate cells in media that contains excess nutrients, and this may directly influence transcriptional response in hypoxia.

**Methods:** We studied the effect of glucose and glutamine concentration on HepG2 hepatoma transcriptional response to low oxygen and expression of hypoxia inducible factor-1α (HIF-1α). Mass spectrometry confirmed low oxygen perturbation of dioxygenase transcripts resulted in changes in DNA methylation.

**Results:** Under normoxic conditions, we observed a significant upregulation of both HIF-target genes and oxygen-dependent dioxygenases in HepG2 cells cultured with physiological levels of glucose or glutamine relative to regular DMEM media, demonstrating that excess glutamine/glucose can mask changes in gene expression. Under hypoxic conditions, *CA9* was the most upregulated gene in physiological glutamine media while *TETs* and *FTO* dioxygenases were downregulated in physiological glucose. Hypoxic regulation of these transcripts did not associate with changes in HIF-1α protein expression. Downregulation of *TETs* suggests a potential for epigenetic modulation. Mass-spectrometry quantification of modified DNA bases confirmed our transcript data. Hypoxia resulted in decreased DNA hydroxymethylation, which correlated with *TETs* downregulation. Additionally, we observed that *TET2* expression was significantly downregulated in patients with hepatocellular carcinoma, suggesting that tumour hypoxia may deregulate *TET2* expression resulting in global changes in DNA hydroxymethylation.

**Conclusion:** Given the dramatic effects of nutrient availability on gene expression, future *in vitro* experiments should be aware of how excess levels of glutamine and glucose may perturb transcriptional responses.

## Introduction

**Figure 1.**
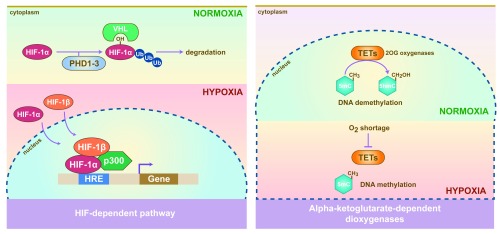
Nutrient availability affects cellular response to stress signals. Cellular response to low oxygen as mediated through HIFs and alpha-ketoglutarate-dependent dioxygenases signalling. HIF-1α is hydroxylated by prolyl-4-hydroxylases 1-3 (PHD1-3) under normoxic conditions and targets the protein for proteasomal degradation by the von Hippel-Lindau (VHL)-E3 ubiquitin ligase complex. Under hypoxia, PHDs are no longer active, allowing s HIF-1α translocation to the nucleus where it heterodimerises with HIF-1β. This heterodimer recognizes and binds to the hypoxia responsive element (HRE), recruits p300 and activates its target genes. The HIF-independent pathway involves the oxygen-dependent dioxygenases. TETs promote the conversion of 5-methylcytosine to 5-hydroxymethylcytosine to achieve demethylation. Under hypoxia, it is thought that TETs promote DNA hypermethylation through a decrease in its activity due to oxygen shortage, resulting in the accumulation of 5-methylcytosine and DNA hypermethylation in tumours.

In most organisms, energy usage is tightly regulated by tissue sensing of nutritional status and the coordination of cellular responses. Oxygen is an essential component in most physiological functions and nutrient availability may influence how cells respond to environments where oxygen is limited. Hypoxia inducible factors (HIFs) are a master regulator of oxygen sensing and define cellular transcriptional response in low oxygen. HIFs activate genes that are essential for cells to survive under low oxygen conditions which helps cells reduce energy demands by limiting oxygen consumption, for instance employing glycolysis in place of oxidative phosphorylation ( Figure 1) ^1^. Another gene family known as the alpha-ketoglutarate-dependent dioxygenases also regulate metabolic homeostasis under low oxygen. They catalyse a range of oxidative processes including hydroxylation and have an absolute requirement for molecular oxygen ^2^. TET dioxygenases are sensitive to changes in oxygen levels and their hydroxylase activities are significantly altered in solid tumours that are characteristically hypoxic ^3^. TET mediates DNA demethylation through the hydroxylation of 5-methylcytosine (5mC) to 5-hydroxymethylcytosine (5hmC), 5-carboxylcytosine (5caC) and 5-formylcytosine (5fC), subsequently followed by base excision repair ( Figure 1) ^4^. Hypoxia-driven changes in TET activity can result in epigenetic alterations that contribute to cancer progression ^1^. 

Hotamisligil *et al*. hypothesized that metabolic systems evolved at a time when modern day pressures such as nutrient surplus were absent or uncommon ^5^. As a consequence, cellular exposure to nutrient excess may alter the magnitude of biological responses and modify metabolic homeostasis, innate immune sensing and inflammatory responses ^6, 7^. Hence, we predict that cellular transcriptional response in hypoxia will be linked to nutrient availability. There is a significant body of literature on the importance of glucose and glutamine concentrations for *in vitro* cell proliferation ^8– 15^. However, relatively little is known about the effect of these nutrients on hypoxia-driven transcriptional responses and epigenetic changes.

The HepG2 human hepatoma cell line is one of the most widely characterized cells in terms of its signalling pathways and transcriptional responses with a large collection of high-throughput functional genomic datasets available in repositories such as Gene Expression Omnibus, ArrayExpress and ENCODE, containing over 1000, 300 and 800 HepG2-based datasets, respectively. However, datasets describing the effect(s) of nutrients on cellular transcriptome have remained scant. Given the widespread use of RNA-sequencing technologies and the current drive to single cell approaches for transcriptional profiling and bio-marker research, the media requirements for any *ex vivo* cell studies are important to consider and standardize. We assessed the effects of media on the transcriptional response of selected HIF-target genes along with oxygen-dependent dioxygenases ^2^. Alterations in DNA methylation levels as a result of oxygen deprivation were also assessed in our preferred media choice.

## Results and discussion

Dulbecco’s Modified Eagle’s medium (DMEM) is one of the most widely used media for culturing cells *in vitro* and contains 25mM glucose and 2mM Glutamax (L-alanyl-L-glutamine), representing 4-5-fold higher concentrations than normal physiological levels. We studied the effect of reducing glucose and glutamine levels to 5mM and 0.5mM respectively on cellular transcription ^12– 17^. HepG2 cells proliferated in media with reduced glucose or glutamine at a comparable rate to those in DMEM and no adverse effects on cell viability were noted.

### Glucose and glutamine concentrations modulate transcript levels of selected HIF-target and dioxygenase genes

Hypoxia and DNA hypermethylation are common in solid tumours, where reduced oxygen levels activate the HIF transcriptional complex ^18, 19^, orchestrating the expression of a wide range of genes in an attempt to restore metabolic homeostasis ^20, 21^. A recent study showed the reduced TET enzymatic activity in hypoxic tumour cells leads to the accumulation of 5-methylcytosine and cancer progression ^3^.

**Figure 2.**
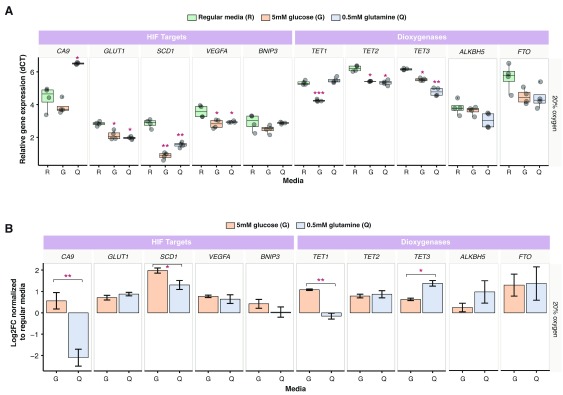
Glucose and glutamine availability affect HepG2 transcript levels under ambient culture conditions. ( **A**) Transcript levels of selected HIF-target genes ( *CA9, GLUT1, SCD1, BNIP3*, *VEGFA)* and dioxygenases ( *TET1, TET2, TET3, ALKBH5* and *FTO*) in HepG2-NTCP cells cultured under ambient oxygen conditions (20%). Data is expressed as normalized values (∆Ct) relative to the internal housekeeping control *Beta-2-Microglobulin (B2M)* (n = 4; error bars ± SEM). ( **B**) Fold induction represented as log2 fold change after normalization of physiological glutamine or glucose values relative to DMEM (n = 4; error bars ± SEM). A Kruskal-Wallis ANOVA with Bonferroni correction was used, p-values were indicated by the following: * < 0.05, ** < 0.01, *** < 0.001. R = regular media, G = physiological glucose, Q = physiological glutamine.

We investigated the effect of culturing HepG2 cells in media with different glucose and glutamine concentrations on transcript levels of several HIF-target genes ( *CA9, GLUT1, SCD1*, *BNIP3* and *VEGFA*) and dioxygenases ( *TETs*, *ALKBH5* and *FTO*) genes under normoxic (20% oxygen) and hypoxic (1% oxygen) conditions. Under normoxic conditions, culturing cells in physiological glutamine or glucose media resulted in a significant change in gene transcript levels compared to cells propagated in DMEM ( Figure 2A). For example, we noted increased mRNA levels of *GLUT1, SCD1, VEGFA, TET2*, and *TET3* in cells cultured in physiological glutamine or glucose compared to DMEM, suggesting that excess nutrients may dampen transcriptional activity. Comparing gene expression data from HepG2 cells cultured in low glucose or glutamine media relative to DMEM showed significant effects on gene expression and highlighted the gene-specific nature of the response ( Figure 2B).

**Figure 3.**
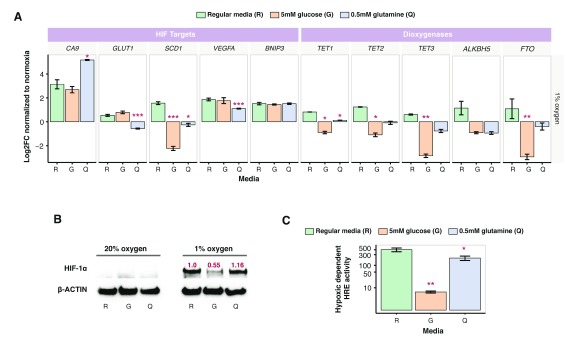
Glucose and glutamine availability affect HepG2 transcript levels under low oxygen. ( **A**) Transcript levels of known HIF-target genes ( *CA9, GLUT1, SCD1, BNIP3*, *VEGFA)* and dioxygenases ( *TET1, TET2, TET3, ALKBH5* and *FTO*) were determined in HepG2-NTCP cells cultured under 1% oxygen for 48h. Hypoxic expression levels were expressed relative to normoxic values (n = 2–4; error bars ± SEM). ( **B**) HIF-1α expression in HepG2-NTCP cells cultured in 20% and 1% oxygen for 48h. Densitometric quantification of HIF-1α expression after normalizing relative to B-actin levels are depicted in red font. ( **C**) HepG2 cells expressing a reporter plasmid encoding a hypoxic responsive element (HRE) driving luciferase expression were cultured under 20% and 1% oxygen for 48h. Luciferase levels under hypoxic conditions are expressed relative to normoxic values (n = 16–24; error bars ± SEM). A Kruskal-Wallis ANOVA with Bonferroni correction was used, where p-values were indicated by the following: * < 0.05, ** < 0.01, *** < 0.001. R = regular media, G = physiological glucose, Q = physiological glutamine.

Exposure of HepG2 cells cultured in DMEM to low oxygen increased the transcription of several HIF-target genes ( *CA9, SCD1*, *BNIP3* and *VEGFA*) and the magnitude of the “hypoxic response” was dependent on the culture media in a gene-specific fashion ( Figure 3A). For example, hypoxic induction of *CA9* was two-fold higher in HepG2 cells cultured in 0.5mM glutamine compared to DMEM, whilst mRNA levels for the *TETs* and *FTO* were significantly reduced in 5mM glucose but not in DMEM ( Figure 3A). This observation is consistent with a recent study reporting reduced TET activity in hypoxic tumour samples that associated with reduced 5hmC measurements in solid tumours ^3^. Taken together, it is likely that excess glutamine/glucose levels perturb the metabolic cues that regulate cellular transcription and cellular responses to stress signals. The breakdown of homeostasis due to nutrient overload leading to aberrant cellular signalling is widely acknowledged to drive a myriad of pathologies such as cancer ^6, 22^, fatty liver disease ^23, 24^ and type 2 diabetes ^25– 27^.

Next, we determined whether the effect(s) of glucose/glutamine levels on HIF-target gene expression was explained by changes in HIF protein expression. HepG2 cells express HIF-1α under low oxygen conditions in all three media types ( Figure 3B), with an approximate 50% reduction in expression in cells cultured in 5mM glucose media ( Figure 3B). In HepG2 cells stably expressing a luciferase reporter under the control of a hypoxia responsive (HRE) promoter, we observed a significant reduction in promoter activity in hypoxic cells cultured in 5mM glucose media ( Figure 3C), consistent with reduced HIF-1α expression. These observations support an association between HIF-1α expression and HRE reporter activity, however, the impact of low glucose or glutamine on HRE activity did not correlate with other host transcriptional responses, demonstrating the simplistic nature of the reporter system. This illustrates the complex nature of HIF interactions with host gene promoter or enhancer elements, where other factors, such as the chromatin and methylation landscape determine the accessibility of any particular region to HIFs.

### Hypoxia regulates epigenetic changes

**Figure 4.**
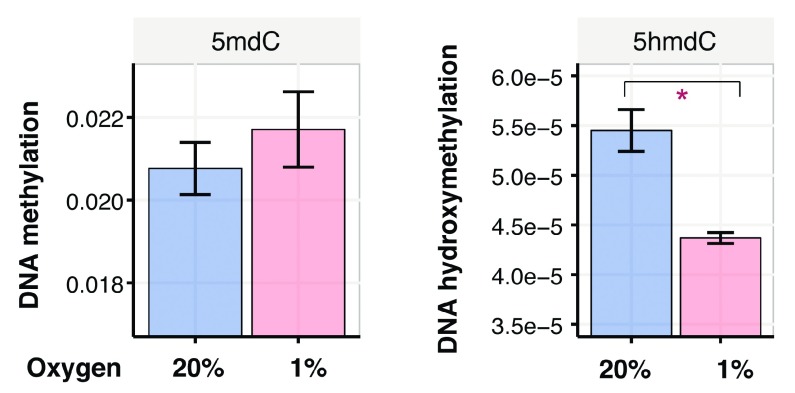
Hypoxia alters HepG2 DNA methylation. Mass spectrometry quantification of DNA methylation (5-methyl-deoxy-cytidine/5mdC) and DNA hydroxymethylation (5-hydroxymethyl-2'-deoxycytidine / 5hmdC) in HepG2 cells cultured in physiological glucose media at 20% and 1% oxygen for 48 hours. A student T-test comparison was used, p-values were indicated by the following: * < 0.05.

We predict that reduced *TET* expression in hypoxia would result in decreased DNA hydroxymethylation and increased DNA methylation. Since we observed a robust downregulation of TETs when HepG2 cells were cultured in 5mM glucose ( Figure 3A), we selected this media of choice for subsequent mass-spectrometry quantification of 5mC and 5hmC. Hypoxic cells showed a modest increase in DNA methylation and a significant reduction in DNA hydroxymethylation ( Figure 4). Low oxygen-dependent reduction of DNA hydroxymethylation is concomitant with a reduction in *TET* gene expression as they catalyse the conversion of 5mC to 5hmC ( Figure 3A). This observation is consistent with another report by Thienpont *et al.* ^3^, suggesting that hypoxia-signalling promotes aberrant DNA methylation and can alter the epigenetic landscape of hepatocyte-specific transcription factors. Of note a recent study reported that low glucose could stabilize *TET2* ^28^. Hence, glucose surplus in regular DMEM could perturb *TET2* expression and may mask any further reduction of 5hmC in hypoxia.

### TET2 expression levels were decreased in hepatocellular carcinoma

**Figure 5.**
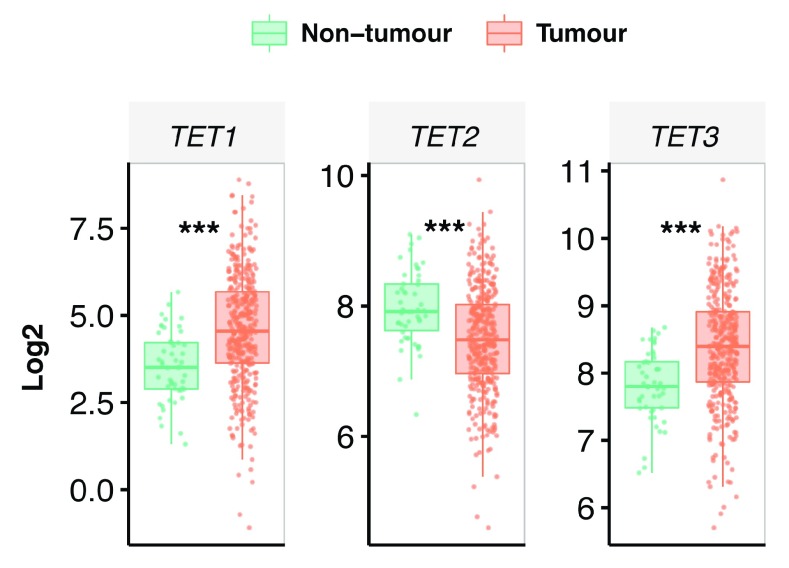
Expression distribution of *TETs* mRNA in hepatocellular carcinoma. Nonparametric Mann-Whitney-Wilcoxon test was used to compare the distribution of *TETs* expression in tumour and adjacent non-tumour samples. Asterisks *** represent P < 0.0001.

Previous studies have demonstrated that oxidative derivatives of 5mC were decreased in hepatocellular carcinoma (HCC) ^29^. This correlated with a significant decrease in *TET2* expression levels and alpha-ketoglutarate content in patients with HCC, suggesting that decrease in TET enzymatic activity resulted in altered expression of DNA methylation enzymes ^29, 30^. Independently, using HCC mRNA expression profiles from The Cancer Genome Atlas, we found reduced *TET2* transcripts in tumour compared to non-tumour samples ( Figure 5). However, *TET1* and *TET3* expression levels were upregulated in tumour samples, suggesting that they may compensate for reduced *TET2* levels in HCC ( Figure 5). Importantly, decreased *TET2* expression in HCC confirmed our earlier observation on hypoxic treated HepG2 cells cultured in physiological glucose media ( Figure 3A and Figure 4).

## Conclusion

We found that a simple change in cell culture media resulted in significant changes in hypoxia transcriptional response ( Figure 2 and Figure 3), occurring through both HIF-dependent and independent pathways. We predict that these changes in mRNA levels will be reflected in protein translation. Moreover, there appears to be global alterations in hepatocellular DNA methylation in hypoxia conditions ( Figure 4) and reduced *TET2* transcript levels in HCC tumours ( Figure 5). We suggest that *in vitro* studies measuring cellular transcriptional responses consider the use of defined media to ensure robust datasets and to promote the translation of *in vitro* datasets to *in vivo* physiological events.

## Methods

### Cell culture

Human hepatoma HepG2 cells were cultured with regular, physiological glucose or glutamine media as follows: regular media is basic DMEM (ThermoFisher Scientific - A1443001) supplemented with 25mM glucose, 2mM glutamax, 10% fetal calf serum (FCS) and 1x penicillin-streptomycin (P/S); physiological glucose is basic DMEM with 5mM glucose, 2mM glutamax, 10% FCS and 1x P/S; physiological glutamine media is basic DMEM with 25mM glucose, 0.5mM glutamax, 10% FCS and 1X P/S. Cells were seeded at 26,000 cells/cm ^2 ^in collagen (Sigma) coated 6-well plates and incubated for 48h under 20% or 1% oxygen (Invivo2, Baker Ruskinn).

### RNA extraction and quantitative real-time polymerase chain reaction (qPCR)

Cellular RNA samples were extracted from normoxic and hypoxic samples using the RNeasy Mini kit (Qiagen) with in-column DNAse digestion performed according to the manufacturer’s instructions. One-step qPCRs for *CA9*, *GLUT1*, *BNIP3* and *VEGFA* were performed using a TaqMan master mix (Takyon, Eurogentec) and TaqMan MGB probes (Applied Biosystems). For detection of *SCD1*, *TET1*, *TET2*, *TET3, FTO* and *ALKBH5* mRNA, cDNA syntheses from 1μg of total RNA were performed using a QuantiTect Reverse Transcription Kit (Qiagen), followed by qPCR using a SYBR green master mix (Applied Biosystems) ( Table S1). The hypoxia insensitive housekeeping gene *Beta-2-Microglobulin* ( *B2M*) was used as an endogenous control. Log2 fold changes were calculated using the 2 ^-∆∆Ct^ method. In order to depict gene expression levels under normoxia, ∆Ct values (Ct gene – Ct *B2M*) were calculated.

### HIF-1α western blotting

HepG2-NTCP cells were seeded at 26,000 cells/cm ^2 ^in collagen (Sigma) coated 6-well plates in the various media and left to adhere overnight. Cells were incubated at 20% or 1% oxygen for 48h and lysed using 8M urea lysis buffer. Approximately 20μg of protein was separated by PAGE and transferred to Polyvinylidene difluoride (PVDF) membrane and incubated with primary antibodies for HIF-1α (BD Biosciences, USA), or β-actin (Thermo Fisher Scientific, USA). Bound antibodies were detected using horseradish peroxidase (HRP) conjugated secondary antibodies (Dako, Agilent Technologies, USA) with chemiluminescent detection (SuperSignal, Thermo Fisher Scientific) using a PXi Touch Gel Imaging System (Syngene).

### HRE reporter assay

HepG2-NTCP cells cultured as described above were lysed and luciferase activity assessed using GloMax luminometer in accordance with the manufacturer’s instructions (Promega). Lysates were quantified for protein concentration using a BCA Protein Assay Kit (Thermo Fisher Scientific) and luciferase activity (relative light units, RLU) expressed relative to protein concentration.

### DNA digestion and HPLC-MS/MS analysis

DNA samples were incubated in hydrolysis solution containing 45 mM NaCl (Invitrogen), 9 mM MgCl _2_ (Ambion), 9 mM Tris-HCl (pH 7.9, Gibco), 25 U Benzonase Nuclease (Sigma-Aldrich), 5 mU Phosphodiesterase I (Sigma-Aldrich), 0.5 μg Alkaline phosphatase (Sigma-Aldrich), 9.36 ng/μL EHNA hydrochloride (Sigma-Aldrich) and 3.52 μM Deferoxamine (Sigma-Aldrich) at 37°C for 4 hours. The digested samples were filtered with Amicon Ultra-0.5 mL 10K centrifugal filters (Merck Millipore) to remove the proteins. The same volume of HPLC buffer A (H _2_O containing 10 mM ammonium acetate, pH 6.0) was added to the filtered samples, and then subjected to HPLC-MS/MS analysis.

The HPLC-MS/MS analysis was carried out with 1290 Infinity LC Systems (Agilent) coupled with a 6495B Triple Quadrupole Mass Spectrometer (Agilent). A ZORBAX Eclipse Plus C18 column (2.1 x 150mm, 1.8-Micron, Agilent) was used. The column temperature was maintained at 40°C, and the solvent system was water containing 10mM ammonium acetate (pH 6.0, solvent A) and water-acetonitrile (60/40, v/v, solvent B) with a 0.4 mL/min flow rate. The gradient was: 0–5 min; 0 solvent B; 5–8 min; 0–5.63 % solvent B; 8–9 min; 5.63% solvent B; 9–16 min; 5.63–13.66% solvent B; 16–17 min; 13.66–100% solvent B; 17–21 min; 100% solvent B; 21–24.3 min; 100–0% solvent B; 24.3–25 min; 0% solvent B. The dynamic multiple reaction monitoring mode (dMRM) of the MS was used for quantification. The source-dependent parameters were as follows: gas temperature 230°C, gas flow 14 L/min, nebulizer 40 psi, sheath gas temperature 400°C, sheath gas flow 11 L/min, capillary voltage 1500 V in the positive ion mode, nozzle voltage 0 V, high pressure RF 110 V and low-pressure RF 80 V, both in the positive ion mode. The fragmentor voltage was 380 V for all compounds, while other compound-dependent parameters were summarized in Table S2.

### HCC RNA-sequencing cohort

The liver cancer dataset (LIHC) used in this study was obtained from The Cancer Genome Atlas (TCGA) ^31^. Gene expression profiles for LIHC were downloaded from the Broad Institute GDAC Firehose ( http://gdac.broadinstitute.org/). RSEM normalized expression profiles were converted to log _2_(x + 1) scale and separated according to TCGA barcodes to tumour and non-tumour categories. Box plots were generated using the R ggplot2.

### Statistical analysis

Statistical analyses were performed using R (version 3.5.1). Details of statistical tests performed can be found in the figure legends.

## Data availability

Raw data for the study, including values for mRNA levels, mass spectrometry measurements, luciferase measurements and raw western blot images are available on OSF: https://doi.org/10.17605/OSF.IO/RXKPW ^32^.

Data are available under the terms of the Creative Commons Zero "No rights reserved" data waiver (CC0 1.0 Public domain dedication).

## Supplementary material

**Tables S1 and S2.** Table S1: List of primer sequences used for qPCR analyses; Table S2: Compound-dependent LC-MS/MS parameters used for nucleosides quantification.
