# Supplementary material for: Glucose and glutamine availability regulate HepG2 transcriptional responses to low oxygen
Source: Wellcome Open Res. 2018 Sep 26;3:126. [Version 1] doi: 10.12688/wellcomeopenres.14839.1 (PMC6178907; doi:10.12688/wellcomeopenres.14839.1)
Supplement: Peer review history

# Peer review: Referee response for version 1

**DOI:** 10.21956/wellcomeopenres.16168.r33994

**Published:** 2018-10-16

**Authors:** Daniel A. Tennant

**Affiliations:** 1 Institute of Metabolism and Systems Research (IMSR), University of Birmingham, Birmingham, UK

The manusript from Lai *et al.* describes an investigation into the role of the supraphysiological levels of nutrients used in conventional tissue culture on *in vitro* investigations into aspects of dioxygenase function and hypoxia-inducible factor (HIF) transcription factor signalling. Overall it is a well-written and there is a clear narrative throughout. The experiments are performed with excellent reproducibility, resulting in data that are easy to interpret. However, there are some over-simplifications in the text and data analysis that result in a degree of over-interpretation at times that could do with resolving.

 Specific points

1. The authors use Glutamax throughout as their means of supplementing glutamine; an alanyl-glutamine dipeptide. It would be beneficial if they could tighten their text to reflect that they are supplementing both amino acids to the medium rather than just glutamine. This may be important as, although not investigated, this additional nutrient may contribute to the phenotype.
2. A further point throughout is that physiologically-relevant concentrations of glucose or Glutamax are supplemented, compared to supraphysiological concentrations of both. Although it is important to observe the effects of reducing each nutrient separately as part of an experimental system, the more relevant condition in physiological terms is the simultaneous reduction in both nutrients. Given that physiological concentrations of glucose and glutamax were shown by the authors to have differential effects in the majority of the data presented, it is not currently clear what the reduction of both nutrients down to physiological concentrations would do.
3. In Figure 1, the authors investigate expression of HIF target genes as well as of some cellular dioxygenases. It would be beneficial, given the later point the authors make (that HIF target gene expression is the product of a number of different activities), to investigate the expression of the dioxygenases that can directly alter HIF expression and activity; PHD1-3 and FIH.
4. The authors rightly point to the link between hypoxia-induced HIF-1α protein expression (Figure 3B) and transcriptional activity (Figure 3C) being complex. However, it is also clear that the 2-fold reduction in overall expression, and what appears to be a 50-fold reduction in reporter activity suggest a significant disconnect between the two. Given that the sub-set of well-described HIF1-target genes are differentially regulated – with most remaining unchanged (CA9, GLUT1, VEGFA and BNIP3) – the data suggest that the reporter is not providing physiologically-relevant information. It is not clear from the Materials and Methods section the nature of the reporter construct utilised – as it is almost certainly derived from a HIF-1α target gene promoter, was it one that was analysed in its endogenous setting (e.g. CA9, GLUT1, VEGFA, etc.)?
5. In the methylation analysis, the authors reported a lower mRNA expression of all three TET enzymes (Figure 3A), and note a decrease in hydroxymethylation of DNA (Figure 4). It should also be noted that no increase in DNA methylation was observed (statistical test fell short of significance), which is therefore in contrast to the aberrant methylation previously reported in hypoxia (reference as cited in the manuscript). Could the authors incorporate a comment on this into the manuscript, or investigate why this is the case? To help support the authors’ further comments on the effects of supraphysiological glucose concentrations on TET2 stability, the protein levels of the TETs could be presented under the conditions investigated. These data would help clarify this somewhat complex issue as it appears that in their system, oxygen concentrations *per se* do not alter global DNA methylation.
6. Finally the authors present data from HCC, where in contrast to the in vitro data presented, the TETs are not apparently co-ordinately regulated (Figure 5 and Figure 3A). It would be useful to clarify this in the manuscript, as the compensation suggested by the authors was not observed in their experimental system.

I have read this submission. I believe that I have an appropriate level of expertise to confirm that it is of an acceptable scientific standard, however I have significant reservations, as outlined above.

# Peer review: Referee response for version 1

**DOI:** 10.21956/wellcomeopenres.16168.r33992

**Published:** 2018-10-08

**Authors:** Jianzhao Liu

**Affiliations:** 1 MOE Key Laboratory of Macromolecular Synthesis and Functionalization, Department of Polymer Science and Engineering, Zhejiang University, Hangzhou, China

This work by Lai *et al.* tells a very interesting story studying the effect of cellular nutrients availability, such as glucose and glutamine, on transcriptional responses to low oxygen. In general, the paper is well written and organized. I have following comments and suggestions:

  

1. Hypoxia inducible factors (HIFs) are a master regulator of oxygen sensing and define cellular transcriptional response to low oxygen level. Under hypoxia, the authors found the nutrients levels including glucose and glutamine, significantly affect the expression of  alpha-ketoglutarate-dependent dioxygenases TETs (TET1, 2, 3), ALKBH5, and FTO.  TETs are DNA demethylases which mediate DNA demethylation through the hydroxylation of 5-methylcytosine to 5-hydroxymethylcytosine (5hmC), 5-formylcytosine, and 5-carboxycytosine, while ALKBH5 and FTO are mRNA N6-methyladenosine (m6A) demethylases. The authors only studied the hypoxia-driven change of TETs expression and the corresponding 5mC and 5hmC alterations in HepG2 cells. I suggest authors look at the mRNA m6A level change because mRNA m6A demethylases ALKBH5 and FTO are also significantly altered under hypoxia. The mRNA m6A modification has been known to post-transcriptionally affect mRNA abundance. The total mRNA expression level changes should arise from both transcriptional (DNA methylation involved) and post-transcriptional (mRNA methylation involved) layers. The investigation on RNA methylation may provide more insights.
2.  In Figure 2A, the axis title ‘dCT’ should be presented as ‘DCt’, otherwise it is misleading.

The trend of TETs expression changes in Figure 3A and Figure 5 show inconsistency. Does it means that cell lines and tumor tissues have different environments?

I have read this submission. I believe that I have an appropriate level of expertise to confirm that it is of an acceptable scientific standard.

# Peer review: Referee response for version 1

**DOI:** 10.21956/wellcomeopenres.16168.r33993

**Published:** 2018-10-01

**Authors:** Bing Yao

**Affiliations:** 1 Department of Human Genetics, School of Medicine, Emory University, Atlanta, GA, USA

How the environmental impact, such as nutritional status and oxygen level, affect intrinsic transcriptome remain less clear. In this study, Lai et al took on this important challenge to demonstrate the nutrition supply and oxygen level in cell culture media play important roles in affecting global transcriptome and DNA methylome. The expression of several HIF target genes and Dioxygenases in HepG2 cells were altered in response to the change of Glucose and glutamine concentrations and oxygen levels in the media. Furthermore, the expression of HIF protein was altered in response to glucose and glutamine availability and positively correlated with its target expression. Importantly, altered dioxygenase expression, including TET proteins, resulted in global change of cytosine modification homeostasis. Finally, they found TET2 expression was also decreased in hepatocellular carcinoma, providing molecular mechanism underlying hypoxia-induced hypermethylation in liver cancer. Recent studies start to shed light on the link between HIF and TET proteins. For instance, HIF1A is significantly upregulated during mast cell activation, in which TET2 plays key roles in shaping epigenetic landscape. However, the roles of HIF-TET axis in response to nutritional status and cancer is not explored. Overall, this is a timely and well-tailored study addressing a fundamental yet under-developed question. The results could offer important molecular insights linking external environmental impact to intrinsic transcriptome and epigenome alteration, and their implications in carcinogenesis.

 Specific comments:

1. The change of gene expression in Figure 2A was presented using dCT. Could the author use log2FC as presented in Figure 2B? These gene expression alterations were assessed under ambient oxygen conditions. Could the author test a few genes under normal DMEM condition?
2. In Figure 3, it is very interesting to demonstrate the change of HIF1a and test its effects using reporter gene bearing HRE, do they have a control reporter gene without HRE?
3. The authors found TET2 is specifically downregulated in liver cancer, which is in line with its established role as tumor suppressor. I am curious whether there are change of HIF1 target genes in these tumor samples?

I have read this submission. I believe that I have an appropriate level of expertise to confirm that it is of an acceptable scientific standard.
